# [^18^F]SynVesT-1 PET Detects SV2A Changes in the Spinal Cord and Brain of Rats with Spinal Cord Injury

**DOI:** 10.2967/jnumed.124.269291

**Published:** 2025-09

**Authors:** Baosheng Chen, Chao Zheng, Tutukhanim Balayeva, Takuya Toyonaga, Xingxing Wang, Jie Tong, William Mennie, Jelena Mihailovic, Daniel Coman, Fahmeed Hyder, Stephen M. Strittmatter, Richard E. Carson, Yiyun Huang, Zhengxin Cai

**Affiliations:** 1 Department of Radiology and Biomedical Imaging, Yale University, New Haven, Connecticut;; 2 Azrieli Centre for Neuro-Radiochemistry, Brain Health Imaging Centre, CAMH, Toronto, Ontario, Canada, and Departments of Psychiatry, Chemistry, Pharmacology and Toxicology, University of Toronto, Toronto, Ontario, Canada;; 3 Departments of Neuroscience and Neurology, Yale University, New Haven, Connecticut;; 4 Department of Biomedical Engineering, Yale University, New Haven, Connecticut; and; 5 Department of Pharmacology, Yale University, New Haven, Connecticut

**Keywords:** spinal cord injury, SV2A, PET, [^18^F]SynVesT-1, simplified reference tissue model 2, diffusion tensor imaging

## Abstract

Traumatic spinal cord injury (SCI) is a devastating neurologic condition lacking effective prognostic and treatment methods. PET imaging of synaptic vesicle glycoprotein 2A (SV2A) has been used in measuring synapse changes. We explore the feasibility of using [^18^F]SynVesT-1 PET to detect the synaptic changes in a rat model of SCI. **Methods:** [^18^F]SynVesT-1 PET scans were performed on rats with T7 moderate contusion injury (*n* = 9) and sham controls (*n* = 7) on day 1 and days 9–11 after injury. The simplified reference region method 2 was used to compute the distribution volume ratios (DVRs) for the spinal cord (SC) and the brain, with the cervical cord and brain stem as the reference region, respectively. The averaged SUV ratio 30–60 min after injection was calculated as an alternative outcome measure. Diffusion tensor imaging (DTI) was used to evaluate axonal changes on post mortem SCs. Western blotting, immunohistochemical staining, and immunofluorescence staining were used to confirm the imaging results. **Results:** [^18^F]SynVesT-1 showed the highest uptake in the cervical SC. Notably, the DVR at the injury epicenter in SCI rats showed a 61% decrease on day 1 and a 53% decrease on days 9–11, compared with sham controls. The changes in SUV ratio 30–60 min after injection were consistent with the changes in DVR. The fiber damage in the epicenter was identified by DTI, and the loss of SV2A was confirmed by immunohistochemical staining and Western blotting. Further, the amygdala, limbic insular cortex, and cerebellum were found to be significantly affected by the SCI on day 1 by PET. The DTI analysis revealed fiber damage in the internal capsule and somatosensory cortex. **Conclusion:** [^18^F]SynVesT-1 PET effectively identified synapse loss in the contusion SCI rat model. The quantification of synaptic density through SV2A PET presents a promising objective metric for evaluating novel therapeutics for SCI.

## Visual Abstract

**Figure:**
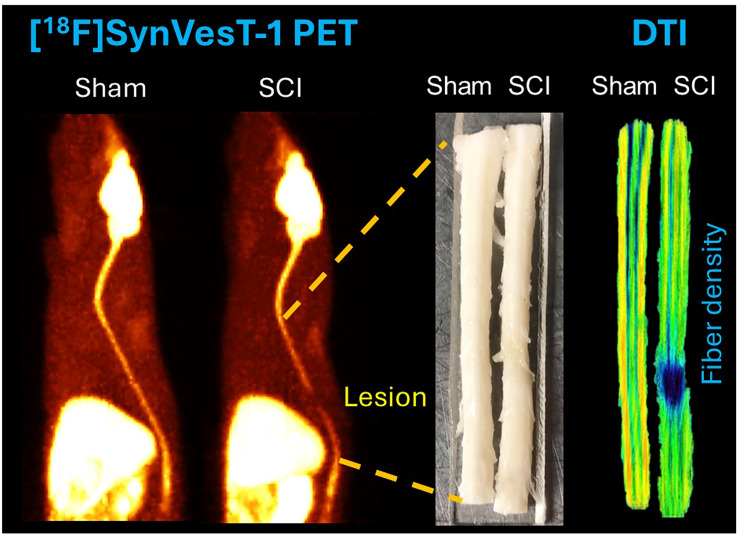

The estimate of annual incidence of traumatic spinal cord injury (SCI) is 54 cases per 1 million people, and approximately 308,600 people in the United States live with SCI (National Spinal Cord Injury Statistical Center). Clinical outcomes vary based on lesion severity and location, potentially leading to partial or complete loss of sensory or motor function below the injury level (1–3), due to damage to the cells, axons, and synapses in the spinal cord (SC) and the brain (4,5).

Current clinical SCI diagnosis relies on anatomic techniques such as x-ray and CT, which assess spinal integrity but provide limited physiologic and pathologic information. Thus, developing noninvasive imaging methods to examine the disease status is crucial. Over the years, several PET radioligands have been explored for SCI, such as [^18^F]FDG, which measures glucose metabolism. However, [^18^F]FDG PET results have not consistently correlated with SC function across disease stages (6–8), potentially due to glial activation (9,10). To overcome this, more specific radioligands targeting translocator protein (11,12), myelin markers (13,14), serotonin transporters (15), and demyelination (16) have been reported. Nevertheless, challenges in clinical translation remain. For example, the serotonin transporter radioligand [^11^C]AFM detected axonal damage in a rodent model of SCI (17) but showed negligible binding in the human SC, likely due to interspecies differences in serotonin transporter density (15).

Recently, synaptic vesicle glycoprotein 2A (SV2A), a 12-transmembrane glycoprotein found in synaptic vesicles of neurons, has emerged as a promising PET imaging target for assessing synaptic density in neurodegenerative conditions, including Alzheimer disease, ischemic stroke (18–21), and traumatic brain injury (22). The temporal and spatial patterns of synapse loss reflected in SV2A PET signals vary across different central nervous system regions and disease states. Since the development of SV2A PET imaging probes (22,23), SV2A PET has been used to assess synaptic density in the SC both preclinically and clinically (15,24,25). Specifically, [^11^C]UCB-J has been tested on a unilateral cervical cord contusion model and showed promising results in identifying the lesion site (24).

In this study, we used the newly developed ^18^F-labeled SV2A radiotracer, [^18^F]SynVesT-1 (20,26–28), to assess changes in synaptic density in a rat model of T7 contusion (17). PET imaging findings were compared with ex vivo diffusion tensor imaging (DTI) and molecular biologic analyses.

## MATERIALS AND METHODS

### Radiochemistry

The radiochemistry procedure to produce [^18^F]SynVesT-1 has been described previously (26) and is briefly outlined in the supplemental materials (available at http://jnm.snmjournals.org).

### Animals

**FIGURE 1.**
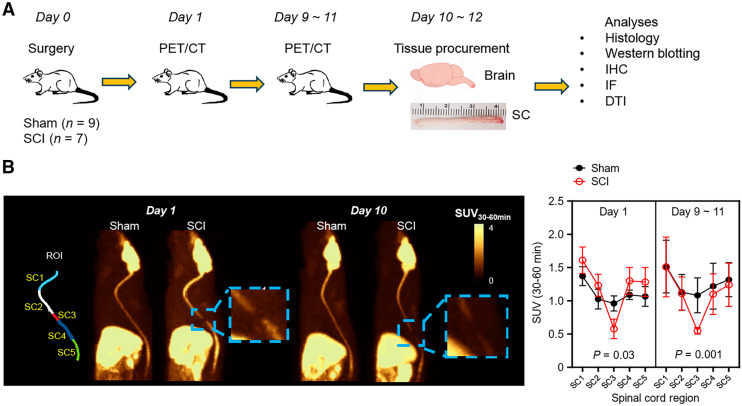
(A) Diagram of experimental design. (B) Left: representative [^18^F]SynVesT-1 PET summed SUV_30–60 min_ images for baseline scans. SC1, C1–C7 (cervical segment); SC2, T1–T6 (upper thoracic); SC3, T7 (lesion epicenter); SC4, T8–T12 (lower thoracic); SC5, L1–L5 (lumbar segment). Right: summed SUV_30–60 min_ of sham controls and SCI rats (*n* = 3–7, multiple *t* tests). IF = immunofluorescence; IHC = immunohistochemistry.

Sprague–Dawley rats (female, 12 wk old, *n* = 16) purchased from Charles River were housed under a 12-h light/dark cycle with ad libitum food and water at the Yale Animal Resource Center. All experiments were conducted with approval from Yale’s Institutional Animal Care and Use Committee. Of the 2 cohorts (16 in total; Fig. 1; Supplemental Table 1), 9 rats underwent SCI procedures at the T7 vertebra using a Multicenter Animal Spinal Cord Injury Study impactor (10 g, 25 mm), as previously described (17), and 7 rats underwent laminectomy surgery as sham controls. Rat bladders were expressed 2 to 3 times daily throughout the study, and cage-side observations were made to monitor overall health and disease progression.

### Western Blotting

Total proteins from spinal segments approximately 0.5 cm rostral and caudal to the injury epicenter were extracted for Western blotting, and the procedure is provided in the supplemental materials.

### Histology, Immunohistochemistry, and Confocal Immunofluorescence

The details of the procedures and the usage of antibodies are provided in the supplemental materials.

### PET Scanning

Sixteen rats underwent baseline or blocking (with levetiracetam) scans using [^18^F]SynVesT-1 on an Inveon PET/CT scanner (Siemens). The first cohort (SCI, *n* = 4; sham, *n* = 4) underwent 1 scan on days 9–11, and the second cohort (SCI, *n* = 4; sham, *n* = 3) underwent 2 scans on day 1 and days 9–11 and was euthanized on days 10–12 with CO_2_ and perfused with paraformaldehyde for postmortem DTI. Five rats (SCI, *n* = 2; sham, *n* = 3) were scanned on days 9–11, with levetiracetam (30 mg/kg, intravenous) preinjected 10 min before tracer injections. Emission data were acquired 60 min after intravenous tail vein injections of [^18^F]SynVesT-1 (18 ± 6 MBq), followed by a 10-min CT scan for attenuation correction and spinal region of interest (ROI) definition.

### Image Analysis

PET images were reconstructed following published procedures (29). To assess SC regions, 5 ROIs (SC 1, 2, 3, 4, 5), including C1–C7 (SC1, cervical), T1–T6 (SC2, upper thoracic), T7 (SC3, lesion), T8–T12 (SC4, lower thoracic), and L1–L5 (SC5, lumbar), each with a diameter of 2.8 ± 0.04 mm (Fig. 1B), were drawn. The volumes of the SC ROIs are provided in Supplemental Figure 2A. Time–activity curves in SUV were generated, and SUVs were normalized to SC1 (C1–C7) to generate SUV ratios (SUVRs). The brain stem (BS) and the whole brain (WB) were explored as alternative reference regions (30). SUVRs from 30 to 60 min (SUVR_30–60 min_) after tracer injection were used to evaluate SV2A levels (29).

For brain PET imaging, the SUV from 0 to 60 min from 1 animal was coregistered with the SIGMA rat brain atlas using FMRIB’s Linear Image Registration Tool (31,32). The coregistered SUV image served as the template for further analysis. Brain regions defined in the SIGMA atlas were analyzed. The simplified reference tissue model 2 (33) was used to estimate the relative tracer delivery rate and distribution volume ratio (DVR), with BS as the reference region. In addition, SUVs of brain regions from 30 to 60 min (SUV_30–60 min_) after injection were normalized by BS to generate SUVR (29), and SC1 and WB were also explored as reference regions to calculate DVR and SUVR.

### DTI and Analyses

The brains and SCs of the rats (SCI, *n* = 4; sham control, *n* = 3) were extracted for ex vivo DTI and analyses. The detailed procedures and analyses for multiple parameters (34), including fractional anisotropy (FA), apparent diffusion coefficient (ADC), mean diffusivity (MD), λ-parallel (λ_ǁ_), and λ-perpendicular (λ_⊥_), are described in the supplemental materials (35).

### Statistical Analyses

All data are presented as mean ± SD. An unpaired Student *t* test was used to compare the difference between the SCI and sham control groups in the experimental outcomes of SUV, SUVR, DVR, ADC, FA, λ_‖_, λ_⊥_, MD, and fiber tractography. Where indicated, 1-way ANOVA was used to compare the difference in the protein expression level among different SC segments and in SUV_30–60 min_ among the sham group and the SCI group at day 1 and days 9–11 after injury. A *P* value of less than 0.05 without correction for multiple comparisons was considered statistically significant.

## RESULTS

### [^18^F]SynVesT-1 PET Imaging Detects SV2A Loss in SC

[^18^F]SynVesT-1 with greater than 99% radiochemical purity was synthesized according to published procedures (27). For each animal, the injected cold mass of SynVesT-1 was 0.08 ± 0.09 µg/kg. [^18^F]SynVesT-1 exhibited SV2A-specific uptake in the SC of both sham control and SCI rats (Fig. 1B), which was blocked by 78% by levetiracetam (Supplemental Figs. 1A and 1B). The heterogeneous tracer uptake along the axis of the SC, with cervical and lumbar regions showing higher signal than the thoracic region (Fig. 1B), indicated uneven expression of SV2A in the SCs. Representative SUV time–activity curves of [^18^F]SynVesT-1 in the SC ROIs of sham and SCI rats on day 1 and days 9–11 are displayed in Supplemental Figures 1C and 1D. In sham rats, the maximum tracer uptake was achieved in all 5 ROIs at approximately 4 min after injection. However, in the SCI rats, the SUV in the lesion epicenter (SC3) peaked at 2 min after injection, whereas the other 4 ROIs reached their SUV_peak_ at about 4 min after injection. There was a relatively slow washout phase in all the SC ROIs during the 30–60 min after injection. The SUVs in SC3 of the SCI group were lower than those in the control group, implicating different kinetics and reduced SV2A density in the SC3 of the SCI rats. The relative tracer delivery rate for SC3 in SCI rats on day 1 was significantly lower than that of the sham control on day 1 (Supplemental Fig. 2B), indicating a reduced blood flow in the lesion region. However, there was no difference in relative tracer delivery rate between the sham and the SCI groups on days 9–11 in SC3, suggesting the blood flow is recovered in SCI rats on days 9–11.

**FIGURE 2.**
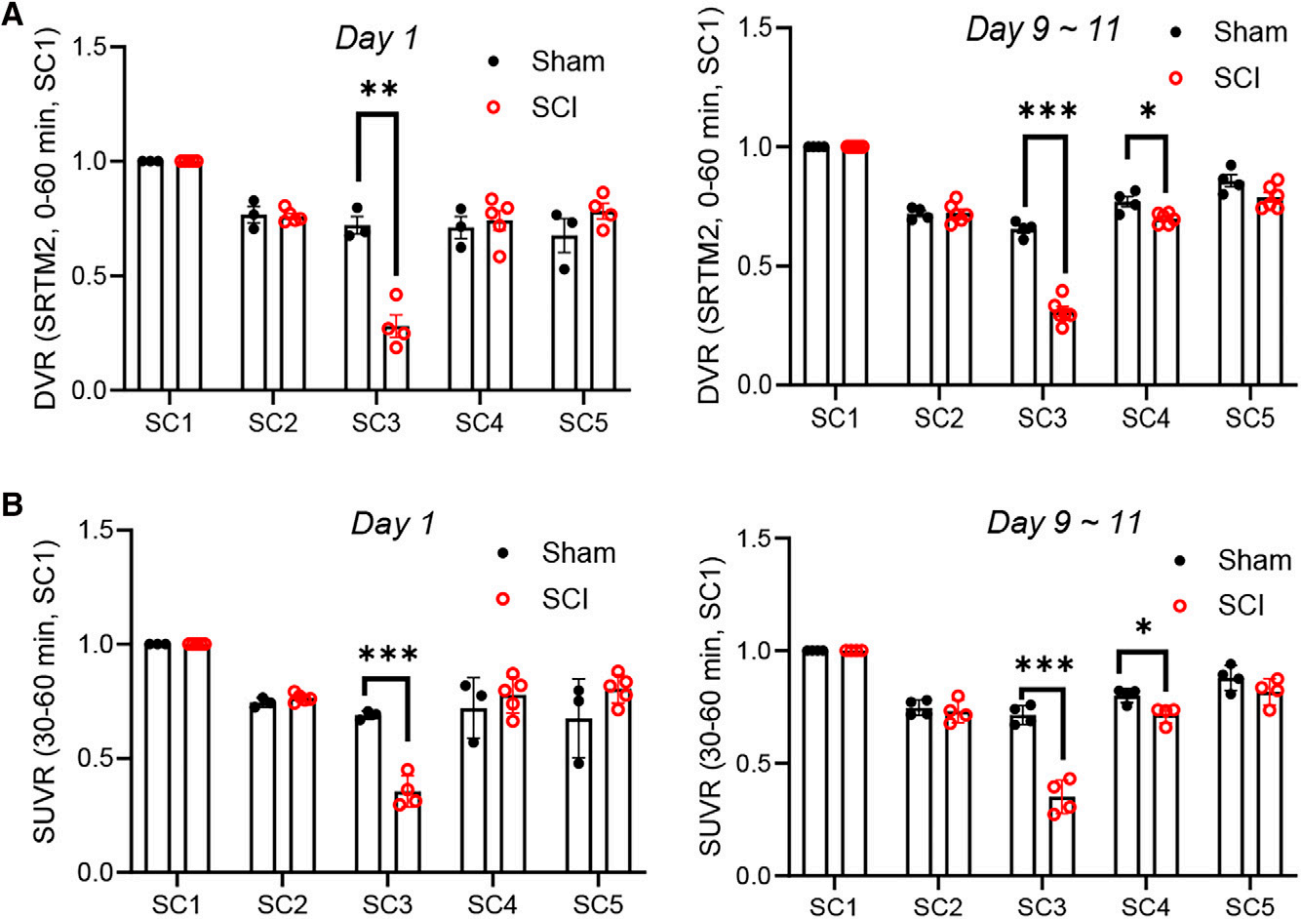
(A) DVRs of [^18^F]SynVesT-1 in different SC regions. Statistically significant differences were observed in SC3 (**P = 0.001, day 1; ***P < 0.0001, days 9–11) and SC4 (*P = 0.01, days 9–11). (B) SUVRs of [^18^F]SynVesT-1 summed from 30 to 60 min after injection. SC3 showed statistically significant differences between SCI and sham group (***P = 0.0005 on day 1 and ***P < 0.0001 on days 9–11). Both DVR and SUVR on days 9–11 show decrease in SC4 (*P < 0.05). *n* = 3–7 for all paradigms, *t* test. SRTM2 = simplified reference tissue model 2.

To calculate the regional DVR, we used simplified reference tissue model 2 with SC1 as the reference region, since SC1 showed similar SUVs in SCI rats and the sham controls (Fig. 1B; Supplemental Figs. 2C and 2D). We observed a 61% lower DVR (sham: 0.72 ± 0.066; SCI: 0.28 ± 0.098; *P* < 0.01) on day 1 in the SC3 region of the SCI rats and a 53% lower DVR (sham: 0.66 ± 0.032; SCI: 0.31 ± 0.052; *P* < 0.001) on days 9–11 (Fig. 2A; Supplemental Table 2).

These differences were consistent when using any of the 3 reference regions (SC1, BS, and WB) (Supplemental Fig. 3). In contrast, the regions rostral (SC1 and SC2) and caudal (SC5) to the epicenter did not exhibit significant differences, except for SC4 in which we observed a small (9%) but significant lower DVR (sham: 0.77 ± 0.043; SCI: 0.70 ± 0.022; *P* < 0.05) on days 9–11 (Fig. 2A) when using SC1 as the reference region. A trend of lower DVR in SC4 on days 9–11 was also seen when using either BS or WB (Supplemental Figs. 3B and 3D) as the reference region.

We next proceeded to calculate the summed SUVR_30–60 min_ imaging window for the SC using SC1 (Fig. 2B; Supplemental Table 3), BS, and the WB (Supplemental Fig. 4) as reference regions. We also performed correlation analysis of SUVR_30–60 min_ and DVR to explore if SUVR averaged 30–60 min after injection was a suitable surrogate for DVR (Supplemental Fig. 5). We found that SUVR_30–60 min_ is well correlated with DVR regardless of the choice of reference region (SC1, *R*^2^ = 0.96; brain stem, *R*^2^ = 0.96; whole brain, *R*^2^ = 0.95).

On both day 1 and days 9–11, SC3 exhibited a 48% (sham, 0.69 ± 0.02; SCI, 0.36 ± 0.07; *P* < 0.05) and 50% (sham, 0.72 ± 0.04; SCI, 0.35 ± 0.08; *P* < 0.05) lower SUVR_30–60 min_ when using SC1 as the reference region (Fig. 2B) and using BS as the reference region (day 1: 48% difference; sham, 0.31 ± 0.003; SCI, 0.16 ± 0.03; *P* < 0.0001; days 9–11: 48% difference; sham, 0.31 ± 0.007; SCI, 0.16 ± 0.03; *P* < 0.0001) (Supplemental Figs. 4A and 4B). Similarly, when using the WB as the reference, SC3 SUVR was lower in the SCI group than in the sham group on both day 1 (44% decrease; sham, 0.30 ± 0.01; SCI, 0.16 ± 0.03; *P* = 0.0003) and days 9–11 (45% decrease; sham, 0.29 ± 0.02; SCI, 0.16 ± 0.03; *P* = 0002) (Supplemental Figs. 4C and 4D).

### Ex Vivo DTI Confirmed the Axonal Damage at the Injury Site in the SC

**FIGURE 3.**
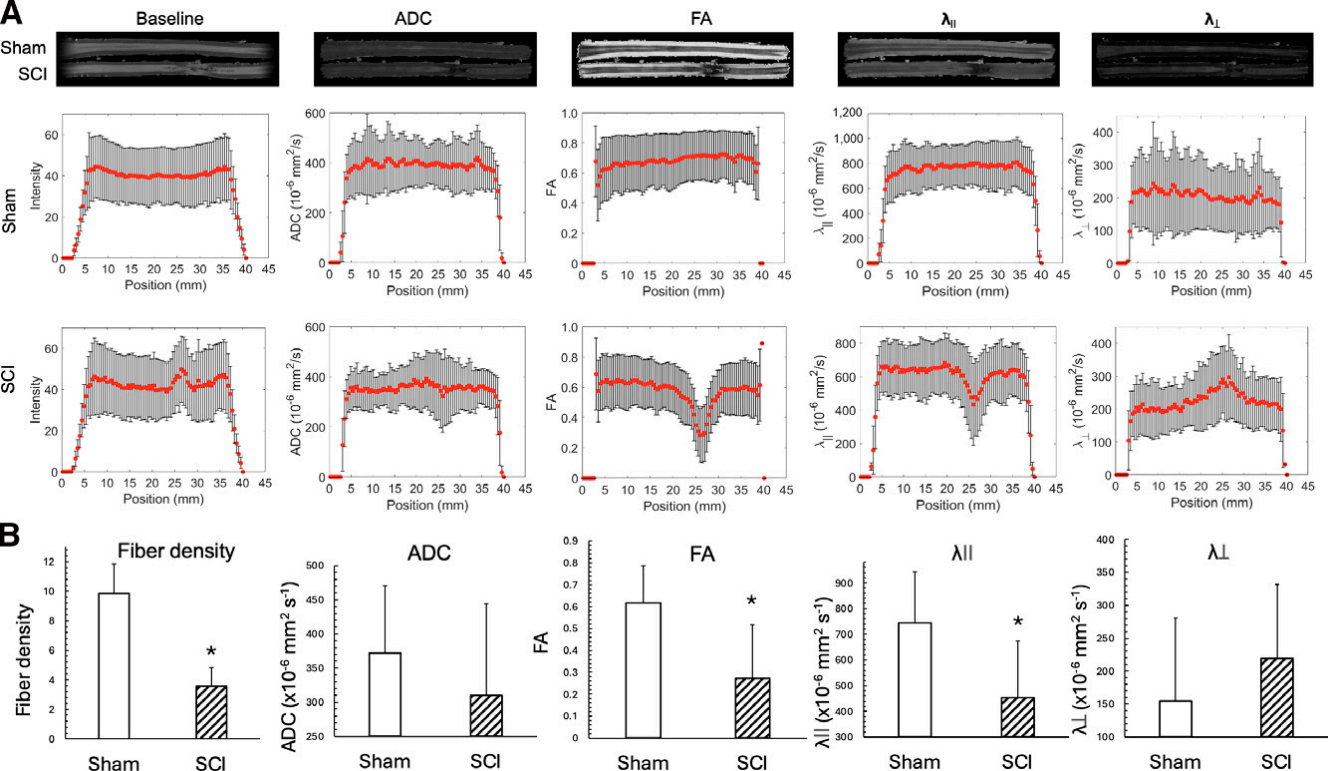
Examples of imaging and quantification of various DTI parameters to assess axonal integrity along SCs. (A) Baseline, ADC, FA, λ_ǁ_ and λ_⟂_ maps of SC segments. (B) Group comparisons of DTI parameters at SCI epicenter and corresponding SC segment of sham controls. SCI, *n* = 4; sham, *n* = 3. *t* test, *P < 0.05.

Representative examples of SC segments (∼3.5 cm) and their MR images in the absence of diffusion gradients (baseline images) and of ADC, FA, λ_ǁ_, and λ_⊥_ parameter maps are shown in Figure 3A and Supplemental Figure 6. Position-dependent analyses revealed significant differences in fiber density, FA, and λ_ǁ_ (Fig. 3B). There was also a slight, but not significant, decrease in ADC along with a slight, but nonsignificant, increase in λ_⊥_. Because FA showed the most significant changes in the injured region, the epicenter of the injury was estimated by determining the position of the lowest FA value along the injured spine (Fig. 3A; Supplemental Fig. 6).

### SV2A Expression Is Decreased in the Injured SC

**FIGURE 4.**
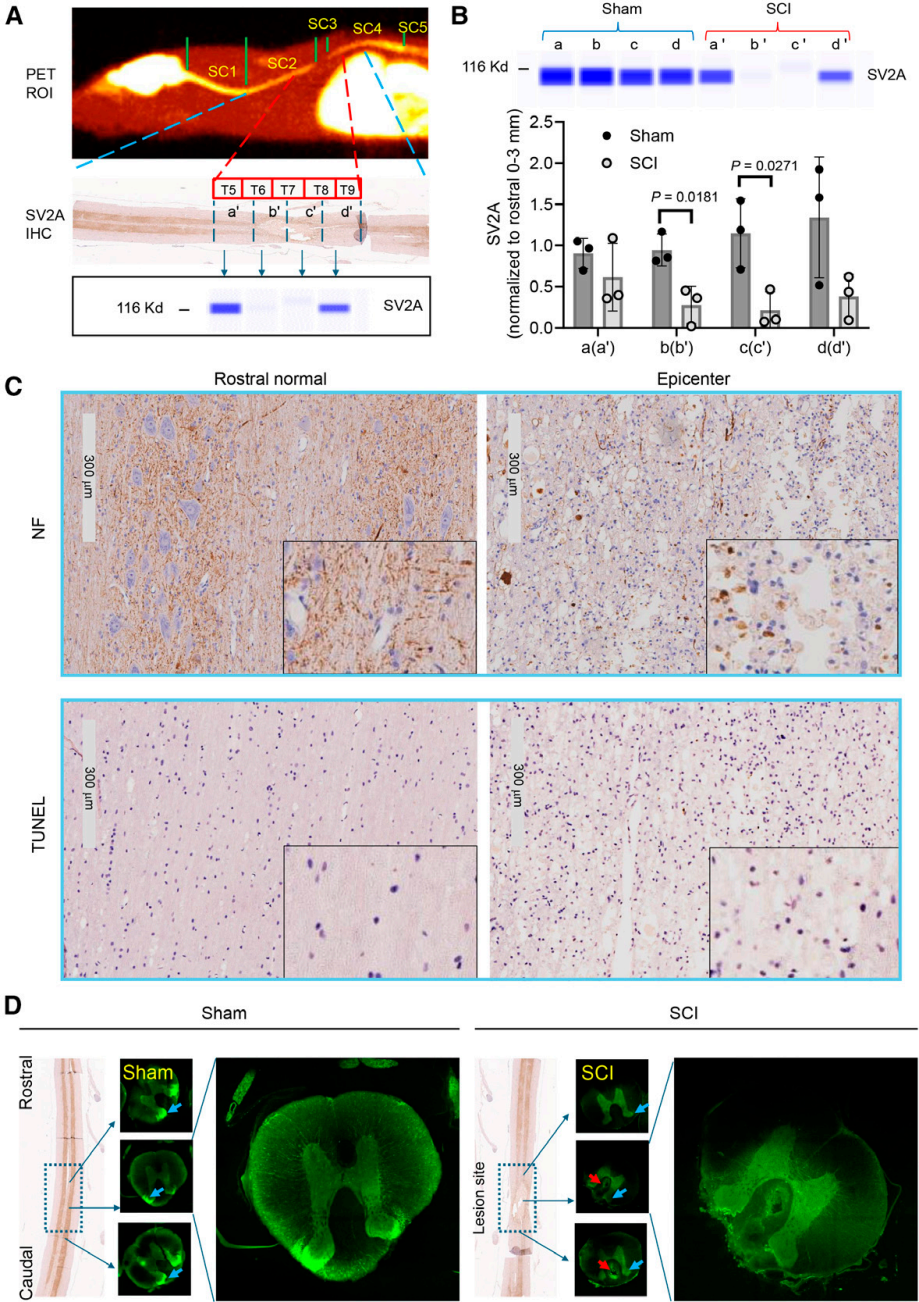
(A) Illustration of tissue sampling sites for immunohistochemical (IHC) staining and Western blotting as correspondent to tissue locations defined using PET SUV images. (B) Representative SV2A capillary Western blotting image and quantification (*n* = 3, *t* test). (C) Top panel: IHC of neurofilament protein (NF) in the normal rostral site (left) adjacent to the epicenter (right). Bottom panel: TUNEL staining shows increased cell death in epicenter. (D) SV2A immunofluorescence staining in transaxial orientated SCs as correspondent to longitudinal IHC. Blue arrows indicate decreased signal in dorsal laminae. Red arrows indicate autofluorescence due to cell death.

The SC (T1–T7) exhibits no visible lesions or scars (Supplemental Fig. 6A). The immunohistochemical staining of SV2A is markedly lower in the epicenter compared with the rostral (T1–T5) and caudal regions (T9–T12) (Fig. 4A; Supplemental Fig. 7). The SC from T5 to T9 was divided into 4 fragments (a′–d′ for SCI; a–d for sham control) as illustrated in Figure 4A for capillary Western blotting. There is no significant difference between the 4 ROIs in the sham control rats (*P* > 0.05, 1-way ANOVA; Fig. 4B). Significantly decreased SV2A expression (∼60% in b′ and 80% in c′) in the SC segments comprising the epicenter was found as compared with the same regions (b and c) in the sham controls. A trend of lower but not significant changes was found in regions of rostral (a′) and caudal to the injury epicenter (d′). The decreased SV2A expression was accompanied by a decreased neurofilament protein (Fig. 4C) and the increased cell death by TUNEL staining at the epicenter of the same SC (Fig. 4C; Supplemental Fig. 8). To examine the synaptic changes transaxially, we performed SV2A confocal immunofluorescence staining in the SC. We found that the level of SV2A staining at the lesion site, specifically in the dorsal horn laminae, was markedly decreased compared with the sham controls as well as at about 2 mm rostral and caudal to the lesion site (Fig. 4D).

### [^18^F]SynVesT-1 PET Imaging Detects SV2A Changes in the Brain

**FIGURE 5.**
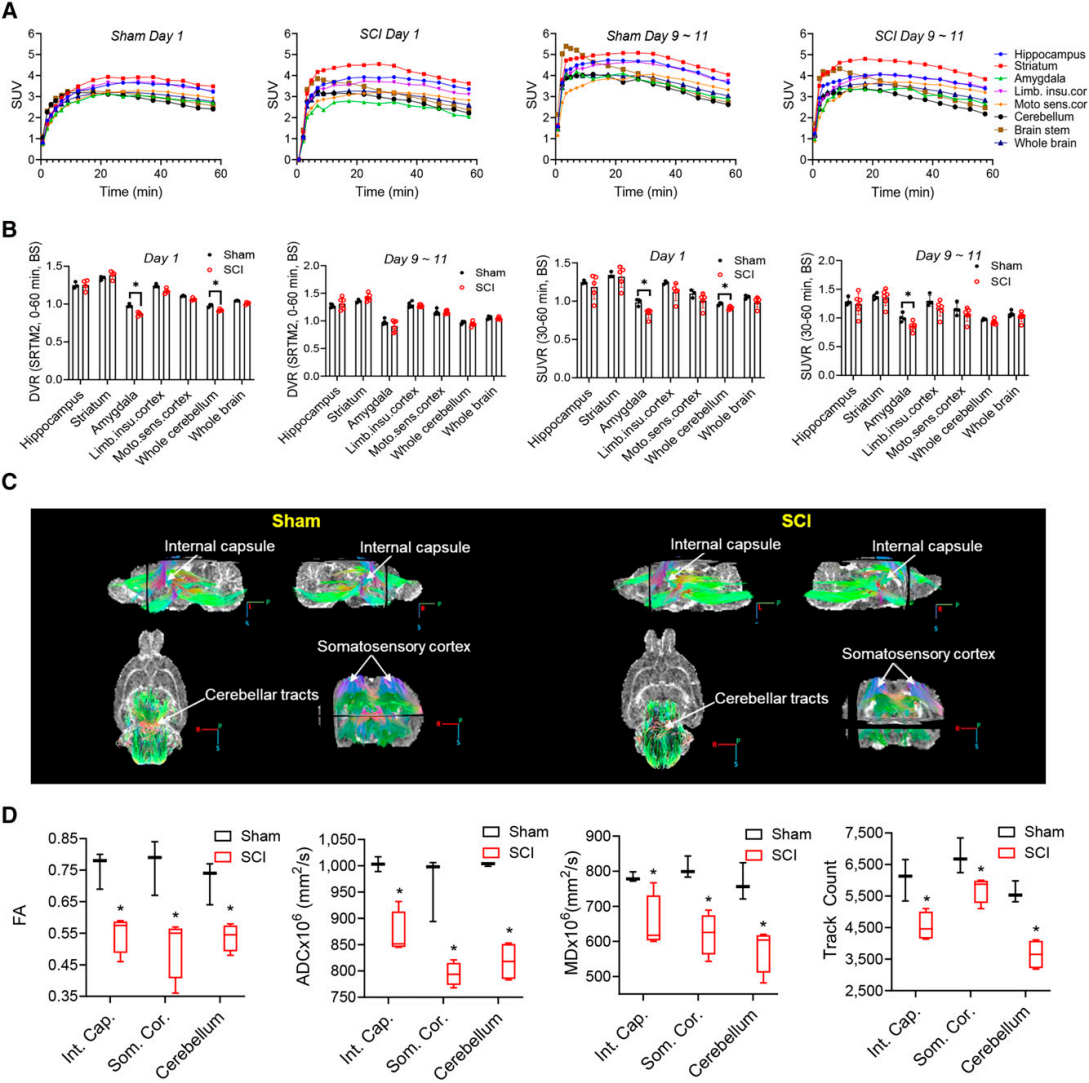
(A) SUV time–activity curves of examined brain regions. (B) DVRs and SUVRs of brain regions. Significantly decreased DVRs were observed in amygdala (*P* = 0.009, day 1) and cerebellum (*P* = 0.046, day 1) in SCI rats compared with sham controls. Amygdala (*P* = 0.03, day 1; *P* = 0.007, days 9–11) displayed significantly decreased SUVRs in SCI compared with sham controls. (C) Representative ex vivo DTI images of SCI and sham rat brains. (D) Voxel-based *t* test comparisons of FA, ADC, mean diffusivity maps, and tractography between SCI and sham groups for internal capsule (Int. Cap.), somatosensory cortext (Som. Cor.), and cerebellum where measured differences were significant. *P < 0.05. Limb.insu.cor and Limb.insu.cortex = limbic insular cortex; Moto.sens.cor and Moto.sens.cortex = motor sensory cortex. SRTM2 = simplified reference tissue model 2.

To assess the impact of SCI on the brain, we extended our analysis to examine the SUV time–activity curves, DVRs, SUVR_30–60 min_, and the summed SUV_30–60 min_ in selected brain regions (Figs. 5A and 5B; Supplemental Fig. 9). There was no difference in the summed SUV_30–60 min_ between the sham control and SCI group in all regions analyzed. However, we found significantly lower DVRs in the amygdala (11%, *P* < 0.05) and whole cerebellum (9%, *P* < 0.05) on day 1 for the SCI group when the BS was used as the reference region (Fig. 5B); however, the DVRs in the amygdala (18%, *P* < 0.01), limbic insular cortex (12%, *P* < 0.05), and WB (9%, *P* < 0.05) were lower on day 1 for the SCI group, compared with the sham group, using SC1 as the reference region. Using WB as the reference region, only DVRs of the amygdala (10%, *P* < 0.05) and motor sensory cortex (2%, *P* < 0.05) were found to be lower on day 1, whereas the DVR of BS on day 1 was slightly but significantly higher (2%, *P* < 0.05) on day 1, compared with the control group (Supplemental Figs. 10A–10D). We next proceeded to calculate SUVR_30–60 min_ for the brain regions. The SUVR in the amygdala was lower in the SCI group on day 1 when any of 3 reference regions was used (Fig. 5B; Supplemental Fig. 11A). Additionally, when SC1 was used as the reference region, the SUVR in the whole cerebellum in the SCI group was significantly lower on days 9–11 (8%, *P* = 0.002) but not on day 1.

The DTI analyses of FA, ADC, MD, and fiber tractography showed that SCI induced significant changes in the internal capsule, somatosensory cortex, and cerebellum regions (Fig. 5D; Supplemental Fig. 12), with changes predominantly occurring bilaterally. No significant difference was found in fiber orientation or density in the thalamic region or cerebral peduncle region.

## DISCUSSION

In this study, we detected synaptic loss at the injury epicenter in a rat contusion model during the acute and subacute phases by using [^18^F]SynVesT-1 PET. Loss in the caudal SC (T8–T12) was detected only during the subacute phase, highlighting the effectiveness of [^18^F]SynVesT-1 in identifying secondary injury-related synaptic loss. We also found a strong correlation between SUVR_30–60 min_ and DVRs, indicating that static imaging can serve as a reliable surrogate of DVR in rats. The axonal damage at the lesion site, assessed through DTI, and significant reductions in SV2A protein levels measured by Western blotting and immunohistochemistry supported the PET results. Additionally, [^18^F]SynVesT-1 detected synaptic changes in the amygdala, cerebellum, and limbic insular cortex in SCI rats.

In our study, we used an established rat T7 contusion model, as consistent spontaneous behavioral and functional improvements were observed over time, with Basso, Beattie, and Bresnahan scores significantly increased from day 10 onward compared with day 1 in previous studies (17). Interestingly, we found the tracer uptake increased slightly on days 9–11 (DVR, −53%) compared with day 1 (DVR, −61%) in the epicenter. Additionally, an increase in Ki-67 protein expression, which is a cell proliferation biomarker, was observed via immunohistochemistry (Supplemental Fig. 13), suggesting possible neurogenesis at the epicenter.

To refine the contusion injury region for PET analysis, we segmented the rat SC into 5 longitudinal ROIs. The tracer uptake was found to be heterogeneous along the SC, aligning with findings in humans and monkeys (36). Notably, SC1 SUV_30–60 min_ were similar in sham and SCI groups, indicating SC1 as a potential reference region for calculating DVR and SUVR for SC analysis. Interestingly, a similar study using [^11^C]UCB-J also used C3 as the reference region for analyzing tracer uptake in their C5 injury model (24). In the study, [^11^C]UCB-J PET was conducted to detect decreased SV2A in the rat SC. They reported reduced signals at both the epicenter (C3) and caudal levels (C6 and C7), which is consistent with our findings of decreased uptake in T8–T12, caudal to the T7 injury site. However, [^11^C]UCB-J has limited clinical potential because of low specific signals in human SC and a short half-life. In contrast, [^18^F]SynVesT-1 has proven useful in various clinical settings and offers better prospects for clinical translation. In addition, we exploratively used BS and WB as reference regions to confirm the decreased DVR and SUVR_30–60 min_ for the epicenter but with no changes in the caudal region (SC4). This highlights the importance of selecting appropriate reference regions for SCI PET imaging analysis.

SCI disrupts communication between the brain and the body, leading to diverse motor, sensory, cognitive, emotional, and autonomic deficits. Significant brain changes occur during the acute to subacute phases of SCI, characterized by inflammation, altered connectivity, neuroplasticity, and potential atrophy. Our PET analysis revealed that the amygdala was the most impacted brain region during these phases, aligning with its function in emotional regulation after SCI. Previous studies showed that SCI directly influences the amygdala through the spino-ponto-amygdaloid pathway, leading to neuroplasticity (37,38). This change may account for the lower SV2A PET signal observed in the amygdala of SCI rats, but further molecular biologic studies are needed to explore the underlying mechanism. The cerebellum exhibited acute phase changes but seemed to adapt over time, showing no alterations in the subacute phase. Collectively, [^18^F]SynVesT-1 PET scans effectively detected synaptic changes in the brains of SCI rats.

Although PET scans indicated synaptic loss in the amygdala from acute to subacute phases, DTI did not identify corresponding fiber changes. This discrepancy may arise because the changes detectable by DTI might occur later than SV2A changes identifiable by PET. Alterations in SV2A levels in the amygdala may manifest early due to stress or shifts in neural connectivity, warranting further investigation. Additionally, DTI may overlook subtle structural alterations that do not significantly impact water diffusion in the amygdala.

Interestingly, whereas SV2A protein levels were lower in the center of the injury, immunostaining indicated that levels were maintained at the periphery, manifesting as aggregated puncta instead of the diffuse staining pattern typically associated with synaptic vesicles. Given the evidence of SV2A expression in mitochondria—especially during the transition from more elongated to fragmented forms under stress—the SV2A immunohistochemistry signals at the injury periphery may be from mitochondria rather than synaptic vesicles (39,40). However, due to the inherent limitations of the partial-volume effect in PET imaging analysis, this low level of preserved signal might not be detectable in PET scans using [^18^F]SynVesT-1. Additionally, the presence of aggregated puncta during cell death often indicates the degradation of proteins with incomplete or misfolded structures which no longer bind to their small-molecule ligands, thus accounting for the absence of [^18^F]SynVesT-1 binding in the injury’s peripheral region.

As a proof of concept, our study has several limitations. First, the limited number of animals may result in some measurements lacking statistical significance. For instance, the amygdala exhibited a trend of decreased DVR in SCI rats on days 9–11 compared with controls, but this difference did not reach significance. Second, although we noted that 6 of 9 SCI animals showed spontaneous locomotor improvement by days 10 and 11, the improvement at the individual level was not tracked. In addition, we did not include male rats in this research. The impact of sex on outcomes has been examined in previous experimental and clinical studies, suggesting a debated sex effect, often indicating better outcomes in females than males (41). Investigating whether SV2A PET imaging can differentiate sex-based variations in diagnosis and prognosis would be a worthwhile future study. Furthermore, we focused solely on comparisons between the acute and subacute phases: including a chronic phase would offer a more comprehensive understanding of disease progression in relation to [^18^F]SynVesT-1 PET imaging. Finally, SCI may compromise the blood–spinal cord barrier, which may lead to tracer metabolites entering the spinal cord and obscure imaging quantification.

## CONCLUSION

Our findings suggest that SV2A is a promising biomarker for SCI. [^18^F]SynVesT-1 PET imaging holds promise as a noninvasive diagnostic and prognostic tool in evaluating SCI progression and monitoring therapeutic effects in preclinical and clinical trials. Although we demonstrated the high sensitivity of [^18^F]SynVesT-1 in detecting the SCI-induced synapse loss at the thoracic region, where the expression of SV2A is the lowest along the SC, its applicability in imaging human SCI still needs to be investigated because of potential species differences in SV2A expression. Using SV2A PET as a surrogate marker for synapse density to predict recovery after SCI remains to be demonstrated and requires further investigation.

## DISCLOSURE

Fahmeed Hyder is the founder of Innovacyclics, LLC. Zhengxin Cai is a cofounder of Synvest Imaging Inc. Richard Carson, Yiyun Huang, and Zhengxin Cai are inventors on PCT/US2018/018388, which covers the SV2A PET tracer used in this study. This research was supported by the National Institute of Neurological Disorders and Stroke of the National Institutes of Health under Award Numbers R01NS123183 and R35NS097283. The content is solely the authors’ responsibility and does not necessarily represent the official views of the National Institutes of Health. No other potential conflict of interest relevant to this article was reported.

## Notes

KEY POINTS **QUESTION:** Is [^18^F]SynVesT-1 sensitive enough to detect synapse loss at the SC injury epicenter and in the brain of an SCI animal model? **PERTINENT FINDINGS:** In the [^18^F]SynVesT-1 PET imaging study, the SUVR at the SCI epicenter was found to be reduced by 58% and 52% on day 1 and days 9–11 after injury, respectively, compared with the sham control rats. The uptake of [^18^F]SynVesT-1 in the amygdala and cerebellum was lower in SCI rats, whereas ex vivo DTI analysis revealed fiber damage in the internal capsule and somatosensory cortex. **IMPLICATIONS FOR PATIENT CARE:** The present findings support the translational evaluation of this imaging method in the clinical diagnosis or prognosis for SCI patients.
